# Reaction dynamics of the methoxy anion CH_3_O^−^ with methyl iodide CH_3_I

**DOI:** 10.1039/d3fd00164d

**Published:** 2024-02-16

**Authors:** Thomas Gstir, David Sundelin, Tim Michaelsen, Atilay Ayasli, Dasarath Swaraj, Jerin Judy, Fabio Zappa, Wolf Geppert, Roland Wester

**Affiliations:** a Institut für Ionenphysik und Angewandte Physik, Universität Innsbruck 6020 Innsbruck Austria roland.wester@uibk.ac.at; b Department of Physics, Stockholm University 106 91 Stockholm Sweden

## Abstract

Studying larger nucleophiles in bimolecular nucleophilic substitution (S_N_2) reactions bridges the gap from simple model systems to those relevant to organic chemistry. Therefore, we investigated the reaction dynamics between the methoxy anion (CH_3_O^−^) and iodomethane (CH_3_I) in our crossed-beam setup combined with velocity map imaging at the four collision energies 0.4, 0.7, 1.2, and 1.6 eV. We find the two ionic products I^−^ and CH_2_I^−^, which can be attributed to the S_N_2 and proton transfer channels, respectively. The proton transfer channel progresses in a previously observed fashion from indirect to direct scattering with increasing collision energy. Interestingly, the S_N_2 channel exhibits direct dynamics already at low collision energies. Both the direct stripping, leading to forward scattering, and the direct rebound mechanism, leading to backward scattering into high angles, are observed.

## Abstract 2

Studying nucleophilic substitution reactions of the polyatomic methoxy anion allows us to narrow the gap to the large systems used in organic synthesis. Despite its complexity, we find predominantly direct dynamics at all studied collision energies.

## Graphical abstract

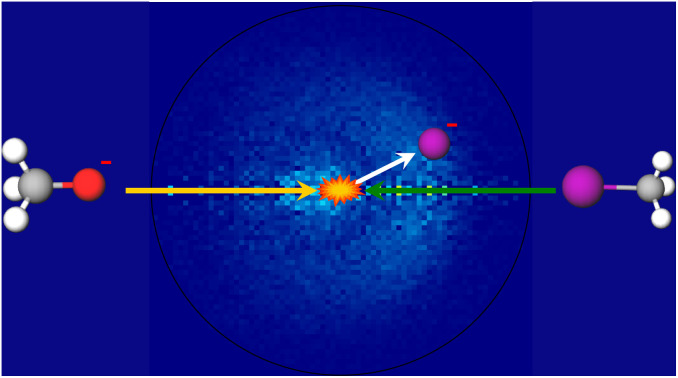

## 1. Introduction

Physical chemistry strives to describe chemical reactions at a fundamental level. To study the reactions free from external influences, gas-phase reactive scattering experiments in crossed-beam setups have been developed.^1,2^ This allows for the precise control of the reactant particles. Of particular interest are the reaction dynamics, which describe the atomic rearrangement during a chemical reaction and help unveil the underlying mechanisms. Measuring differential cross sections (DCS) is one way to unravel the dynamics. In early experiments, rotatable detectors were used to reconstruct the DCS from measurements of the products’ angle and kinetic energy.^3^ This technique is limited since it can only measure a small fraction of product ions at a time, and slow product ions are not efficiently detected. The development of the velocity-map-imaging technique (VMI)^4^ allowed for the construction of more efficient setups.

Ion–neutral reactions constitute a particularly intriguing class of chemical processes. Due to the long-range interaction between the charge of the ion and the neutral and the associated large scattering cross-sections, these reactions play a crucial role in interstellar chemistry and in the ionosphere. Here, charge transfer reactions^5–13^ and studies of the growth of carbon chains^14^ can help us to understand how more complex molecules have evolved. Similarly, catalytic reactions rely on charge transfer *via* ions, and model systems can elucidate the underlying mechanisms.^15,16^ Organic chemistry is a different field where reactions involving ions play a significant role. Most synthesis processes occur in the liquid phase, where solvated ions react with neutral molecules. To study the influence of the liquid environment, microsolvation experiments, where a finite number of solvent molecules are added to the reactant ion, can be conducted.^17–21^

A prominent reaction mechanism in organic synthesis is the nucleophilic substitution (S_N_2) reaction. The simplest system where this reaction can take place is the attack of an anion on a methyl-halide: X^−^ + CH_3_Y → Y^−^ + CH_3_X. Here, the anion X^−^, called the nucleophile, substitutes for the leaving group Y^−^ at the carbon atom. An example of these processes in organic synthesis is nucleophilic substitution on alkyl halides by alkoxy anions, a common pathway to synthesize ethers, referred to as the Williamson ether synthesis.^22^ The potential energy surface of this reaction family in the gas phase is comprised of a double-well structure, where a central barrier separates the pre- and postreaction complexes.^23^ The typical description of the S_N_2 reaction involves the nucleophile attacking the central carbon in a colinear fashion from the opposite side of the leaving group. This leads to the formation of a pre-reaction complex exhibiting *C*_3v_ symmetry. However, extensive studies of this system have found different possible entrance channels and thus revealed a richness in associated reaction mechanisms.^24–26^ The various mechanisms manifest themselves as distinct scattering features in the differential cross-sections (DCS). An attack from the carbon side, called a back-side attack, leads to an inversion of the stereocenter, known as the Walden inversion.^27^ Theoretical calculations have shown a front-side attack with no such inversion to be possible.^28^ The experimental observation of a front-side attack is, however, challenging since it is expected to result in indirect scattering. The “classic” collinear approach leads to the direct rebound mechanism, manifesting as direct backward (the direction of the initial ion) scattering in the DCS. Forward-scattering is indicative of a direct stripping mechanism, where the incoming nucleophile abstracts the CH_3_ moiety at a large distance, barely influencing the trajectory of the product ion.^29^ A low energy feature in the backward direction has been attributed to the roundabout mechanism.^30,31^ For reactants with two or more carbon atoms, significantly more complex reaction dynamics occur that require detailed theoretical calculations to be disentangled.^32,33^

This study presents our findings on the reaction between methoxide (CH_3_O^−^) and methyl iodide (CH_3_I). Hereby, we strive to bridge the gap from simple model systems to larger organic molecules, with CH_3_O^−^ being the largest molecular nucleophile we have studied so far. Methoxide is the conjugate base of methanol, acting as both a strong base and good nucleophile.^34^ It is one of the simplest alkoxides, and together with sodium or potassium, it forms organic salts widely used in organic synthesis processes,^35^ like the above-mentioned Williamson ether synthesis. One of its applications is as the primary catalyst in biodiesel production.^36^ Given its essential role in synthesis processes, understanding its reaction dynamics and pathways is paramount.

In the title reaction CH_3_O^−^ + CH_3_I we find two reaction pathways:

1 CH_3_O^−^ + CH_3_I → I^−^ + CH_3_OCH_3_ Δ*H* = −2.7 eV  (S_N_2)

2 CH_3_O^−^ + CH_3_I → CH_2_I^−^ + CH_3_OH Δ*H* = 0.2 eV (PT)

The listed reaction enthalpies Δ*H* are based on the standard enthalpies of formation at 0 K.^37^ For CH_2_I^−^, the value is taken from our previous calculations.^38^ In the next section, a brief description of the experimental setup and the applied methods is given. The subsequent section presents the experimental results based on the reactive scattering experiment. This is followed by their discussion and a comparison to other similar systems. In the conclusion, the findings for this system are summarized.

## 2. Methods

The reactive scattering experiments were performed on a crossed-beam setup, described in more detail previously.^39^ To create the reactant CH_3_O^−^ we evacuate a mixing gas tank to approximately 10^−2^ mbar and open it to a reservoir containing liquid methanol. Due to a relatively high vapour pressure of 130 mbar, vaporized methanol travels into the gas tank until an equilibrium is reached. Thereafter, we add 8 bar argon to achieve the desired methanol concentration. The resulting gas mixture is then supersonically expanded with a backing pressure of 3 bar into a vacuum chamber. The ionic species is created in a plasma discharge, *via* dissociative electron attachment. A Wiley–McLaren-type spectrometer is employed to extract the ions perpendicular to their initial flow direction, and a set of electrostatic lenses and deflectors are used to guide them into an octupole RF trap. There, the ions are collisionally cooled to room temperature by a buffer gas. We compared the usage of helium and nitrogen due to the expected higher cooling efficiency of the latter, based on the better mass ratio and higher polarizability, to identify potential differences in the scattering results. After 40 ms, the ions are extracted from the trap and accelerated into the scattering chamber. At its center, the ions collide with the neutral beam. The neutral beam is created by expansion of methyl iodide mixed with helium into the vacuum chamber. To avoid clustering of CH_3_I molecules, we only use a weak supersonic expansion, leading to low-speed ratios and less efficient cooling. While methyl iodide is a liquid at standard conditions, its vapour pressure (175 mbar) is high enough to allow for the analog mixing procedure as with methanol. To achieve the desired concentration of CH_3_I in the beam and to prevent clustering, 5 bar helium is added on top of the methyl iodide. The backing pressure used in the expansion of the neutral beam is 0.7 bar. Following the crossing of the ions and neutrals and their reactive interaction, the products are extracted perpendicular to the interaction region and mapped with an optimized velocity map imaging spectrometer^40^ onto the detector, where both position and time of impact are recorded. The position allows us to calculate the velocities (*v*_*x*_ and *v*_*y*_) parallel to the detector surface. From the flight time we can both infer the mass and the out-of-plane velocity (*v*_*z*_) of an ion.

To acquire a complete kinematical picture of the reactive collision, the velocity vectors of all involved particles have to be known. To this end, the average velocity vectors of the incoming ion, incoming neutral, and the 3D velocity vector of the ionic product are measured. The missing vector of the outgoing neutral can be determined by applying the conservation principles of energy and momentum. While the ionic reactants can be directly measured by selecting the appropriate extraction time, the neutral reactant beam has to be ionized before extraction using electron impact. Additionally, the velocity and angular spread of the beams are recorded for error calculation. With this information, the 3D velocity distribution of the products can be transformed into the center of mass reference frame. Due to cylindrical symmetry along the collision axis, the obtained differential cross-sections are represented on a 2D plane with the velocity components parallel (*v*_*x*_) and perpendicular 
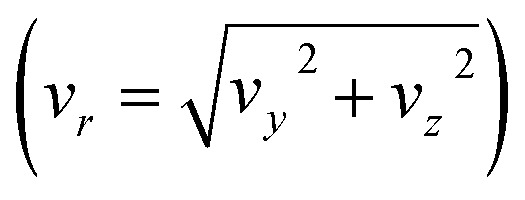
 to the collision axis. The velocities are weighted with 1/*v*_*r*_ to have the images represent a slice through the 3D distribution. The direction of the ionic reactant defines the positive *x*-direction.

To quantify the different reaction mechanisms, the scattering features associated with these are separated by applying a combination of velocity and angular cuts. For the S_N_2 channel, ions with velocities smaller than 25% of the kinematic cutoff are attributed to the indirect mechanism, as illustrated in Fig. 3. Those with velocities greater than that and scattering angles *θ* up to 40°, representing a cone in the forward direction with an open angle of 80°, are assigned to forward scattering. For the direct rebound mechanism, ions in the backward hemisphere and outside of the indirect cut are used. The remaining part in the forward hemisphere is assigned to sideways scattering. Similarly, for proton transfer, ions with velocities smaller than 25% of the maximum attained velocity are attributed to an indirect mechanism, and those with velocities greater than that and falling within a 100° cone in the forward direction are counted as forward scattered (see Fig. 5). For the associated errors, the limits of the velocity cuts are varied by ±10%. The branching ratios for the different reaction channels are calculated using the amount of ions associated with the respective channels. The counting statistics errors are below 1% and are therefore not given explicitly.

## 3. Results

**Fig. 1:**
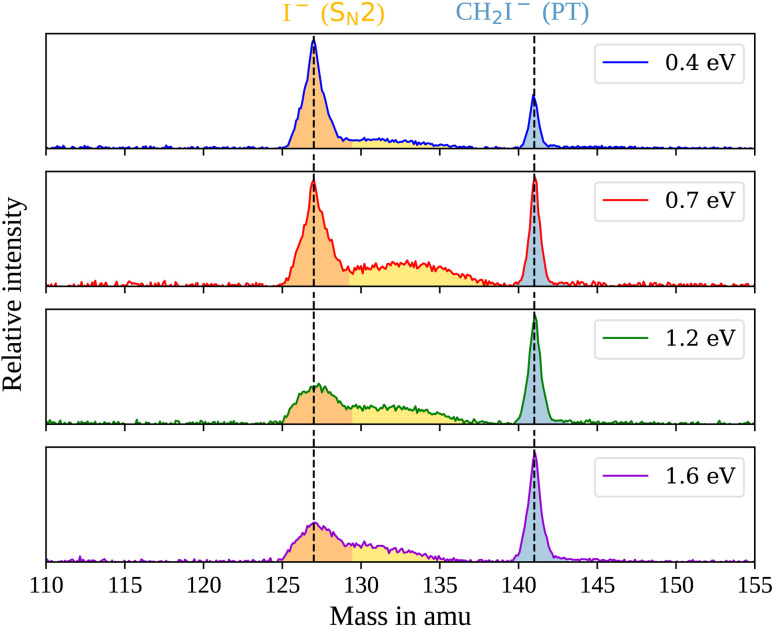
Mass spectra for the reaction CH_3_O^−^ + CH_3_I at the four measured collision energies. The shaded areas mark the mass range associated with the product I^−^ stemming either from a fast mechanism (orange) or a long-lived collision complex (yellow) and CH_2_I^−^ (blue). The vertical lines mark the flight time of ions with an out-of-plane velocity (*v*_*z*_) of zero.

**Fig. 2:**
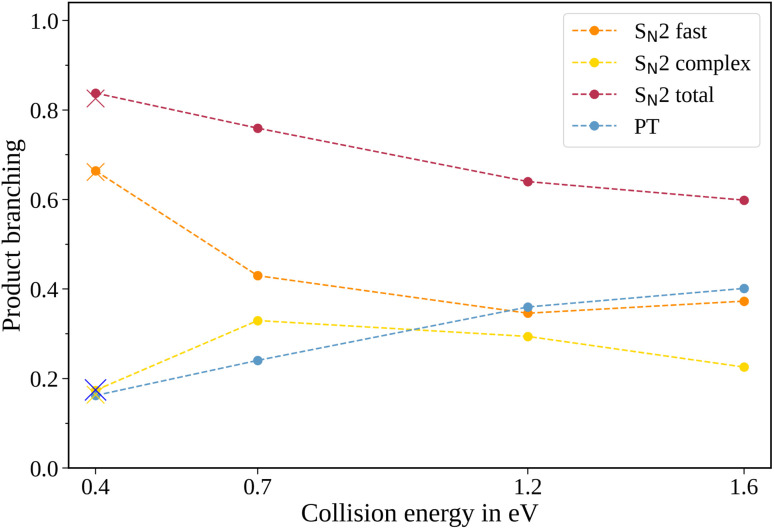
Product branching ratios for the two pathways in the reaction CH_3_O^−^ + CH_3_I. In addition to the total S_N_2 signal, the individual contributions of the peak and tail of the mass spectra are given. The crosses show the branching ratios for the experiment conducted with N_2_ instead of He as a buffer gas at 0.4 eV collision energy. The uncertainties are smaller than the size of the data points.

In the measured reaction of methoxy anions with methyl iodide, we observe the products I^−^ and CH_2_I^−^. These products can be attributed to the nucleophilic substitution (S_N_2) and proton transfer (PT) reaction channels, respectively. In the mass spectra in Fig. 1, the peaks associated with the iodide ions are accompanied by a broad tail extending to higher masses at all collision energies. We have observed an analogous feature in the micro-hydrated S_N_2 reaction F^−^(H_2_O) + CH_3_I → I^−^ + CH_3_I + H_2_O.^20^ These tails stem from the dissociation of long-lived CH_3_O^−^(CH_3_I) complexes after extraction into the VMI spectrometer. At first, an entire complex is accelerated, resulting in an initially lower velocity. However, as it breaks apart, the ion’s mass is reduced, leading to higher acceleration. Consequently, an iodide ion originating from one of these complexes exhibits a longer flight time than a bare ion produced by the fast substitution mechanism, resulting in a higher apparent mass. Depending on the individual lifetime of a complex, different flight times are observed, producing the tails in the mass spectra. For the branching ratio in Fig. 2, the contributions of the peak, tail, as well as the total S_N_2 signal are considered. The individual components correspond to the shaded areas in the time of flight traces.

The contribution of the S_N_2 channel to the total product ion signal decreases from 83% at the lowest to 62% at the highest collision energy while staying the dominant channel. Conversely, the formation of iodide ions through the long-lived collision complex, the tail in the mass spectra, exhibits an initial increase from 0.4 to 0.7 eV followed by a subsequent decrease. Simultaneously, relatively more CH_2_I^−^ ions, formed in the proton transfer channel, are detected, with their contribution increasing from initially 16% to 40%. This trend of the proton transfer channel gaining importance with increasing collision energy has been observed previously in related systems.^41^

**Fig. 3:**
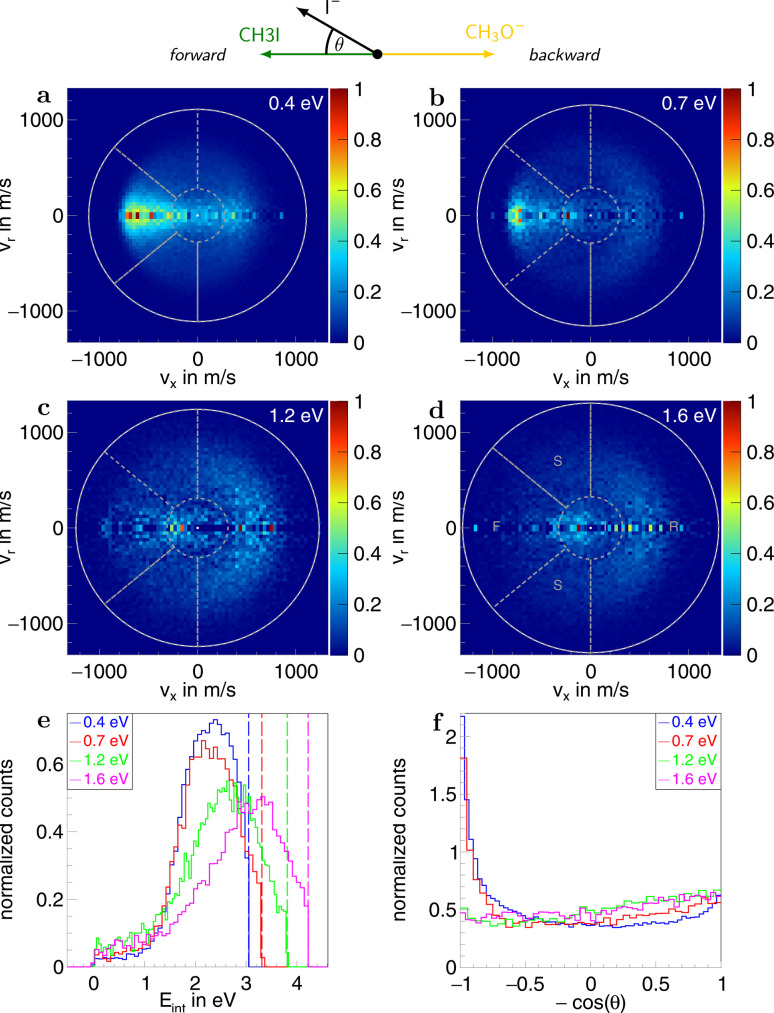
(a–d) Normalized two-dimensional representation of the 3D center of mass velocity distributions of the product ion I^−^ stemming from the S_N_2 pathway of the reaction CH_3_O^−^ + CH_3_I at the four collision energies (a) 0.4 eV, (b) 0.7 eV, (c) 1.2 eV and (d) 1.6 eV. The white superimposed rings give the maximum attainable velocity (kinematic cut-off). The grey dotted lines show the cuts applied to isolate the different scattering mechanisms: F (forward scattering), I (indirect), R (direct rebound), and S (sideways scattering). (e) Normalized internal energy distributions of the product ions. The vertical lines give the maximum available internal energy. (f) Normalized angular distributions of the product ions. At the top, a schematic representation of the beam orientations in the center of mass frame is given. The forward direction is defined by the incoming neutral and, consequently, the backward direction by the incoming ion. The angle *θ* is the angle between the product ion and the incoming neutral, called the scattering angle.

Information on the dynamics is obtained from the product ion scattering images. The S_N_2 channel exhibits direct dynamics already at low collision energies. This behaviour is evident in the differential cross-sections (Fig. 3a–d), where most of the visual flux is located away from the center, which marks the point of zero velocity and, thus, maximum internal excitation of the products. A decrease in the scattering signal is observed towards the kinematic cut-off, constituting the maximum attainable velocity for the products, given the collision energy and the reaction enthalpy. Therefore, some kinetic energy is transformed into rovibrational excitation of the neutral product CH_3_OCH_3_. This is to be expected since, in such a large molecule, an abundance of vibrational modes is available, and the methyl group rotations can be excited as well. The direct scattering behaviour is visible in a more quantitative way in the internal energy distributions of the products, which peak below their maximum available energy (see Fig. 3e), indicating that only a small amount of energy is transferred into internal degrees of freedom. The direct scattering behaviour persists across the measured energy range.

**Fig. 4:**
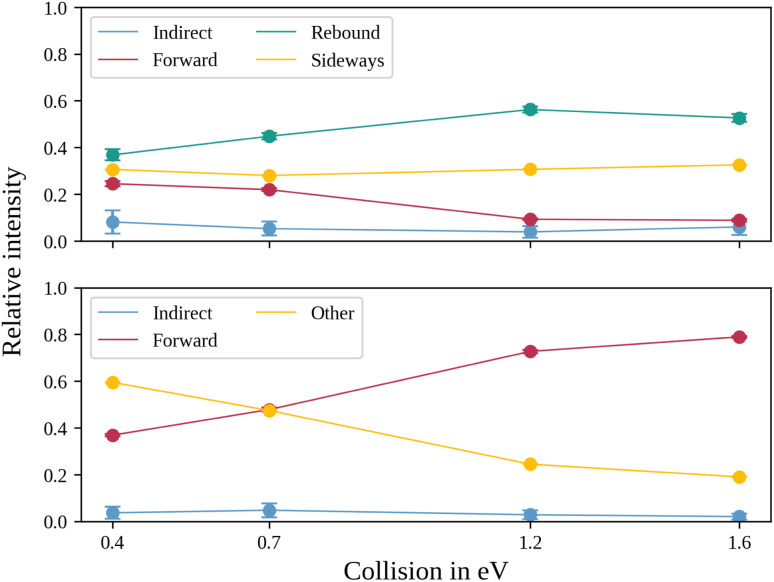
Normalized ion yields for the different reaction mechanisms for the S_N_2 (upper panel) and the proton transfer channel (lower panel). The values are obtained by a combination of different angular and velocity cuts, described in more detail in the method section.

In the upper panel of Fig. 4, the relative contributions to the total scattering signal of the individual mechanisms observed in the S_N_2 reaction are given. Even though the formation of a substantially long-lived complex is observed, indirect scattering plays a subordinate role, with a maximum contribution of 8(5)% at the lowest collision energy. The differential cross-sections and the angular distributions (Fig. 3f) give insight into the scattering direction. At the lowest collision energy, 24(1)% of the iodide ions are formed by a stripping-like mechanism, leading to direct forward scattering. However, the scattering behaviour is dominated by the direct rebound mechanism, leading to the well-known umbrella feature in the backward direction.^30^ Its contribution increases from 37(2)% at the lowest to 53(2)% at the highest collision energy. Additionally, sideways scattering into the forward hemisphere plays a significant role in all measured energies.

**Fig. 5:**
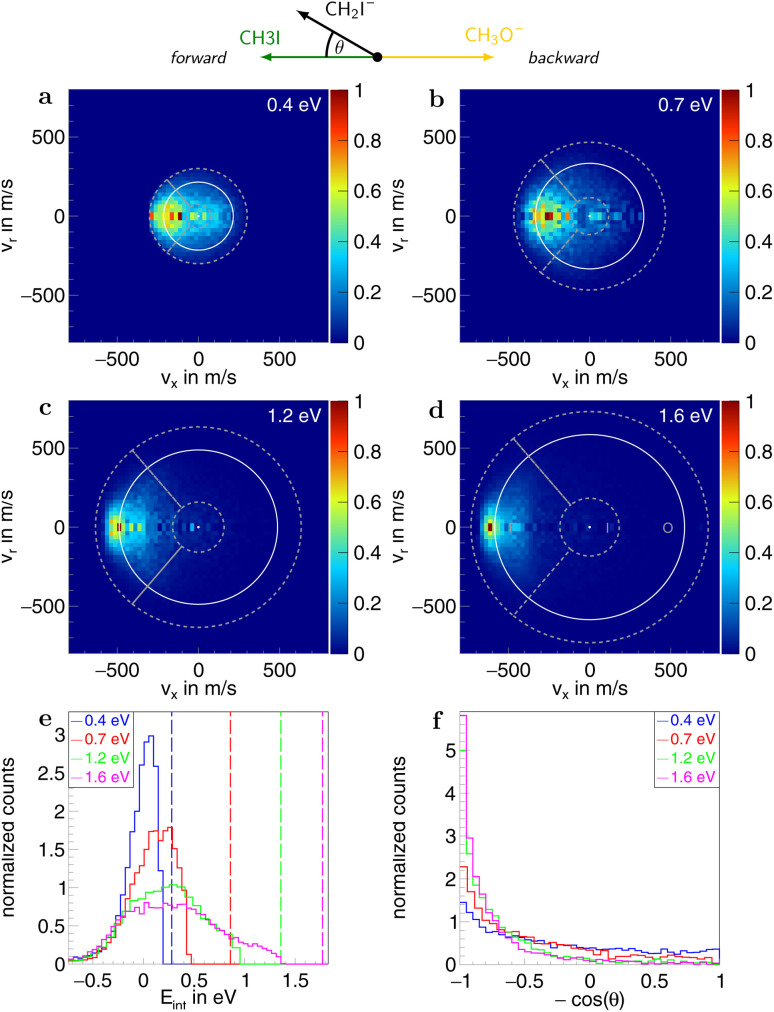
(a–d) Normalized two-dimensional representation of the 3D center of mass velocity distributions of the product ion CH_2_I^−^ stemming from the proton transfer pathway of the reaction CH_3_O^−^ + CH_3_I at the four collision energies (a) 0.4 eV, (b) 0.7 eV, (c) 1.2 eV and (d) 1.6 eV. The white superimposed rings give the maximum attainable velocity (kinematic cut-off). The grey dotted lines show the cuts applied to isolate the different scattering mechanisms: F (forward scattering), I (indirect), and O (other). (e) Normalized internal energy distributions of the product ions. The vertical lines give the maximum available internal energy. (f) Normalized angular distributions of the product ions. At the top, a schematic representation of the beam orientations in the center of mass frame is given. The forward direction is defined by the incoming neutral and, consequently, the backward by the incoming ion. The angle *θ* is the angle between the product ion and the incoming neutral, called the scattering angle.

In a similar fashion to the S_N_2 channel, the lower panel of Fig. 4 shows the relative contributions of the forward and indirect mechanisms in the proton transfer channel. The dynamics are already highly direct at low collision energies with only 4(3)% indirect scattering. Direct forward scattering becomes more prevalent, increasing from 37(1)% to 79(1)% of the product ions formed in this process. This trend is evident in the differential cross-sections, as illustrated in Fig. 5a–d. Accordingly, the energy distributions peak increasingly away from the maximum available energy, meaning lower internal excitation of the products and, therefore, higher kinetic energy (see Fig. 5e). The angular distributions also show the shift towards increasing forward-scattering (Fig. 5f).

In polyatomic reactants, excited vibrational levels could influence the chemical reaction. In previous studies of CH_3_I we found the vibrational excitation of the C–H stretching modes to have an effect on the dynamics and branching ratios of the reaction.^42–44^ The lowest vibrational mode in CH_3_I corresponds to the C–I stretch, with a characteristic wavenumber of 533 cm^−1^,^45^ equivalent to a temperature of 767 K. Given the estimated temperature of around 150 K in the weak supersonic expansion forming the neutral beam, we expect most molecules to populate the vibrational ground state. The methoxide is, however, produced in a plasma discharge, and we rely on buffer gas cooling in the ion trap to quench excited modes. The vibrational frequencies of the CH_3_O^−^ ion all lie above 1000 cm^−1^ (ref. 46) and are therefore frozen out after cooling to room temperature. Apart from the temperature of the buffer gas, its cooling efficiency is critical. Consequently, we compared the scattering results when using either helium or molecular nitrogen as a buffer gas. The branching ratios for the N_2_ case at 0.4 eV collision energy are represented as crosses in Fig. 2 and closely align with those obtained using helium (represented as dots), in agreement with full thermalization in the ion trap.

**Fig. 6:**
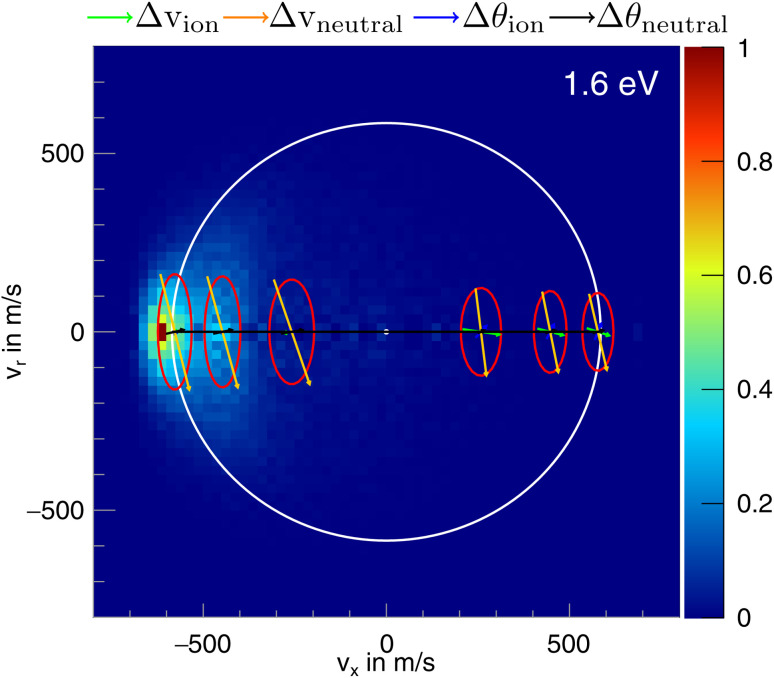
Normalized two-dimensional representation of the 3D center of mass velocity distributions of the product ion CH_2_I^−^ stemming from the proton transfer pathway of the reaction CH_3_O^−^ + CH_3_I at 1.6 eV collision energy. The superimposed ring shows the maximum attainable velocity. The red ellipses depict the r.m.s. (1*σ*) velocity uncertainty at the specific location. The arrows inside the ellipses show the contribution of the uncertainty in velocity of the incoming ion (Δ*v*_ion_) and neutral (Δ*v*_neutral_) and their respective errors in angle (Δ*θ*_ion_ and Δ*θ*_neutral_), the colors of the arrows are shown above the image.

In the differential cross-sections of the proton transfer, a significant portion of the product ions is situated outside the kinematic cutoff, an effect observed before for proton transfer.^43^ This can be similarly seen in the internal energy distributions, which extend to negative energies. We attribute this divergence to the finite energy resolution of our experiment and the increase of the cross section for this channel with collision energy. In Fig. 6, the scattering image at 1.6 eV is overlayed by ellipses, illustrating the uncertainty in product ion velocity at several points along the collision axis. The four contributing 1*σ* uncertainties are in the incoming ion velocity (Δ*v*_ion_), in the incoming neutral velocity (Δ*v*_neutral_), and in their angular spread Δ*θ*_ion_ and Δ*θ*_neutral_. At the kinematic cut-off, the velocity uncertainty in the *x*-direction amounts to 162 m s^−1^ r.m.s. In this case, the largest contributing factors are the uncertainty in angle and velocity of the neutral beam. Furthermore, ions in the high energy part of the reactant energy distribution are subject to a larger proton transfer cross section, in particular close to the threshold for this channel, which adds a shift to larger product ion velocities besides broadening the measured distributions.^43^ With this the signal outside the kinematic cutoff can be well explained.

## 4. Discussion

The dynamics in the S_N_2 channel depend sensitively on the characteristics of the attacking nucleophile. To illuminate their specific influence, we have extensively studied reactions of the form Y^−^ + CH_3_I. For the attacking anion Y^−^, we have used chloride (Cl^−^),^32,47^ cyanide (CN^−^),^48^ fluoride (F^−^),^42,44^ hydroxide (OH^−^),^17^ and the oxygen anion (O^−^).^49^ These different systems have highlighted the importance of the minimum energy structure in the entrance channel for the dynamics of the S_N_2 reaction. In the reaction Cl^−^/CN^−^ + CH_3_I, most product signal in the S_N_2 channel is observed as an umbrella shape in the backward direction of the differential cross-sections. This scattering feature has been attributed to the direct rebound mechanism and is linked to a carbon-bonded entrance channel (Cl^−^/CN^−^⋯CH_3_I) characterized by *C*_3v_ symmetry.^50,51^ With F^−^ and OH^−^ as the attacking nucleophiles, we additionally observe strong indirect and direct forward scattering. The indirect mechanisms arise from an initial hydrogen-bonded complex with *C*_S_ symmetry on the entrance channel side of the potential energy surface.^52–54^ Direct forward scattering is indicative of a stripping mechanism.

Given the size of the methoxide nucleophile, direct dynamics were not expected at low collision energies in the reaction with CH_3_I. Nonetheless, direct stripping, leading to forward scattering, and the rebound mechanism, leading to scattering into high angles in the backward hemisphere, contribute significantly to the total signal. With higher collision energies the latter becomes increasingly dominant. Therefore, we expect that the reaction proceeds predominantly *via* the carbon-bonded entrance channel. Precise alignment of the nucleophile and the neutral reactant is necessary for the collinear approach leading to the rebound mechanism. Indirect scattering plays a subordinate role, further supporting this assumption. Despite being composed of five atoms the methoxide nucleophile therefore reacts quite similarly to chloride or cyanide reactants. Even larger nucleophiles will be needed to explore how and where the reaction dynamics change in the transition to complex polyatomic organic molecules.

The second channel we observe in the studied reaction is the proton transfer channel, where the incoming ion abstracts a proton forming methanol (CH_3_OH) and the CH_2_I^−^ ion. The dynamics of this channel progress from highly indirect, complex-mediated to direct forward, stripping-like with an increase in collision energy. This transition is well known from proton transfer in other ion–neutral reactions.^41,43,54–57^ At low collision energies, a collision complex is formed that is stable for at least one rotation, leading to a statistical distribution of the products. Furthermore, it allows for efficient energy transfer into internal degrees of freedom, resulting in low kinetic energies of the products. With increasing kinetic energy of the reactants, the complex formation becomes increasingly unlikely since the excess energy cannot be efficiently transferred to internal excitation. The reaction transitions towards a stripping-like mechanism, where the incoming ion seizes one of the protons of the neutral at a large distance, barely influencing its initial trajectory, leading to forward scattering of the CH_2_I^−^ product.

## 5. Conclusion

In a number of previous studies, we investigated S_N_2 dynamics of atomic or diatomic anions. Here, we widen our understanding of the attacking anion’s role in the S_N_2 reaction by studying the larger molecular anion CH_3_O^−^. We report on the scattering behavior and branching ratios of the reaction CH_3_O^−^ + CH_3_I at the four collision energies 0.4, 0.7, 1.2, and 1.6 eV. Differential cross-sections were obtained utilizing a crossed-molecular beam setup paired with a 3D-VMI spectrometer. From these, we extract angular distributions and, together with the calculated exothermicities, internal energy distributions of the products. We find the two reaction channels, S_N_2 and proton-transfer, leading to the ionic products I^−^ and CH_2_O^−^ respectively. The proton transfer channel progresses in a well-known fashion from indirect to direct scattering and gains in importance with increasing collision energy. The S_N_2 channel exhibits more intricate dynamics, with highly direct scattering across the measured collision energy range. At the lower energies, a significant fraction of the products are scattered in the forward direction due to a stripping-like mechanism. The direct rebound mechanism becomes increasingly important at higher collision energies, resulting in an umbrella-like shape in the differential cross sections. From comparisons with other previously studied systems, we conclude that the reaction transpires *via* the carbon-bonded entrance channel.

With the present study, we push the investigation of S_N_2 reaction dynamics further to larger molecules, which are important in organic chemistry. Interestingly, the measured differential cross sections still resemble the reaction dynamics observed for atomic chloride or diatomic cyanide nucleophiles. In future studies, even larger nucleophiles and reactant molecules are conceivable, from larger alkane or alkene chains to aromatic hydrocarbons. In parallel, recent experimental developments, such as photoionization ion sources^5^ and higher resolution crossed-beam setups^58^ will open up further opportunities for better resolved differential cross-section measurements.

## Conflicts of interest

There are no conflicts to declare.
